# Aromatic molecules on low-index coinage metal surfaces: Many-body dispersion effects

**DOI:** 10.1038/srep39529

**Published:** 2016-12-22

**Authors:** Yingda Jiang, Sha Yang, Shuang Li, Wei Liu

**Affiliations:** 1 Nano Structural Materials Center, School of Materials Science and Engineering, Nanjing University of Science and Technology, Nanjing 210094, Jiangsu, China

## Abstract

Understanding the binding mechanism for aromatic molecules on transition-metal surfaces in atomic scale is a major challenge in designing functional interfaces for to (opto)electronic devices. Here, we employ the state-of-the-art many-body dispersion (MBD) approach, coupled with density functional theory methods, to study the interactions of benzene with low-index coinage metal surfaces. The many-body effects contribute mostly to the (111) surface, and leastly to the (110) surface. This corresponds to the same sequence of planar atomic density of face-centered-cubic lattices, *i.e.*, (111) > (100) > (110). The binding energy for benzene/Au(110) is even stronger than that for benzene/Ag(110), due to a larger broadening of molecular orbitals in the former case. On the other hand, our calculations show almost identical binding energies for benzene on Ag(111) and Au(111), which contradicts the classic *d*-band center theory that could well predict the trend in chemisorption energies for various small molecules on a number of metal surfaces. Our results provide important insight into the benchmark adsorption systems with opener surfaces, which could help in designing more complex functional interfaces.

Hybrid inorganic-organic systems (HIOS) are the building blocks for many (opto)electronic devices, such as photovoltaics, light-emitting diodes, and molecular sensors and switches^1^,^2^,^3^. The hybrid interfaces often consist of aromatic molecules and transition metal surfaces^4^,^5^,^6^,^7^. Among these, closest-packed metal surfaces, in particular the (111) surface of face-centered cubic (FCC) lattices, were most extensively used, due to the low energy, high stability, and easy processability^8^,^9^,^10^,^11^. However, for realistic applications the non-closest-packed surfaces are sometimes evitable and can be used in, for example, enantioselective separators, heterogeneous catalysis, and field-emitter displays^12^,^13^,^14^,^15^. As recently reported by Eren *et al*.^16^, during catalytic reactions even small molecules, *e.g.* CO, can decompose the closest-packed Cu(111) surface into disordered structures and enhance the reactivity of surface for water dissociation. In this regard, a complete understanding of the interaction of molecules with more open, and even “imperfect” structures, such as steps, kinks, impurities, and defects, is highly demanded for practical uses^17^,^18^,^19^,^20^. However, due to the complex local atomic structure, which is accompanied by the highly corrugated potential-energy surface^13^, it remains a tremendous task to reliably predict the structure and energetics for molecules on non-closest-packed surfaces.

To date, experimental investigations of benzene on (110) and (100) surfaces of coinage metals are sparse, although several theoretical studies have focused on these systems. However, density-functional theory (DFT) calculations often overestimate the adsorption distance and underestimate the binding energy, due to the lack of the long-range vdW interactions for nonhomogeneous electron densities. Recent studies have well noticed the crucial role of vdW interactions in hybrid systems^4^,^21^,^22^,^23^,^24^. However, most of the available vdW-inclusive methods neglect the pronounced contributions of many-body electronic correlations, which arise from the collective electron excitations in the system^25^.

Recently, a promising method named many-body dispersion (MBD) was developed to go beyond the pairwise correction scheme^26^. This method computes the long-range correlation energy through the coupled harmonic oscillator model Hamiltonian, which is an effective random phase approximation-like treatment of many-body effects^27^,^28^. The DFT+MBD method has been demonstrated to perform very well for supramolecular systems, molecular crystals, nanoclusters, and layered nanostructures^25^,^29^,^30^,^31^,^32^,^33^. For adsorption systems, some of us have recently shown that this method can predict the structure and stability of the benzene/Ag(111) system within experimental error bars^33^. However, to the best of our knowledge, the role of many-body contributions in more open surfaces have not been systematically studied yet.

To better understand the nature of bonding for aromatic molecules on low-index metal surfaces, here we systematically study the adsorption of benzene on the (110), (100), and (111) surfaces of Cu, Ag, and Au. Our calculations show that the MBD effects play a prominent role for all studied systems, reducing the binding energies by at most 0.26 eV for Cu(111) compared to the data from the pairwise DFT+vdW^surf^ method^34^. The contribution of MBD effects in different systems is closely related to their corresponding planar atomic density of FCC metals, which is defined as the fraction of total crystallographic plane area that is occupied by atoms. In addition, the binding energy for benzene/Au(110) is even stronger than that for benzene/Ag(110), which somewhat against our intuition that Ag surfaces are more reactive (or at most equal stable) than Au surfaces. This finding can be well explained by extent of broadening and splitting of the molecular orbitals of the adsorbate near the Fermi level. Consistent with experimental results^33^, we find close binding energies for benzene on Ag(111) and on Au(111), due to the delicate balance between Pauli repulsion and vdW forces. Given to the fact that the *d*-band center of Au(111) is significantly closer to the Fermi level than that of Ag(111), our observations seem to contradict the classic *d*-band center theory of Hammer and Nørskov^35^.

## Results

**Figure 1:**
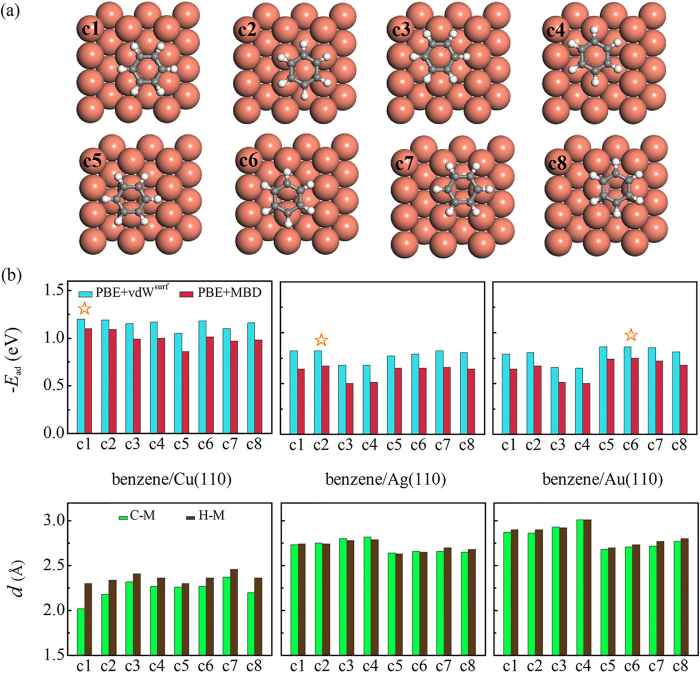
(**a**) Top views of eight high-symmetry adsorption structures for benzene on the (110) surface of coinage metals. Red, grey, and white denotes copper, carbon, and hydrogen atoms, respectively. (**b**) Binding energies and adsorption distances for benzene on the (110) surface of Cu, Ag, and Au from the PBE+vdW^surf^ and PBE+MBD methods. The most stable structure for each system is indicated by five-pointed star.

We first explored the potential-energy surface (PES) for benzene at the (110) surface – the most open surface – of Cu, Ag, and Au. Figure 1a shows the eight high-symmetry starting geometries including four adsorption sites (“hollow”, “atop”, “short bridge”, and “long bridge”) and two orientations of C-C bond relative to the substrate (“perpendicular” and “parallel”). For geometry relaxations, we used the pairwise DFT+vdW^surf^ method^34^, combined with the semi-local Perdew-Burke-Ernzerhof (PBE)^36^ functional. Based on the relaxed geometries, we further employed the MBD method^25^,^26^ for single-point energy calculations. The adsorption energy, *E*_ad_, of benzene on metal surfaces was determined by,


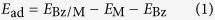


where *E*_Bz/M_, *E*_M_, and *E*_Bz_ denotes the total energy of the adsorption system, the relaxed bare metal slab, and the relaxed gas-phase benzene, respectively. For more details on methods and computation settings refer to Methods section.

Different from the flat PES for benzene/Cu(111)^22^, Fig. 1b shows a larger energy corrugation in benzene/Cu(110) from the PBE+MBD method (0.24 eV). The PBE+MBD adsorption energies are consistently smaller than those from PBE+vdW^surf^, originating from the dynamic screening effects included in the many-body calculations. For benzene/Cu(110), the c1 structure is the most stable from both PBE+MBD (−1.10 eV) and PBE+vdW^surf^ (−1.20 eV). Note that, the PES for benzene on Cu(110) has also been investigated, which shows that the c2 is the most preferable site for benzene on Cu(110)^37^. Besides energetics, the different nature of bonding in three systems can also be evidenced by their adsorption geometries. As shown in Fig. 1c, for benzene on Cu(110) at c1, the *d*_C–M_ is significantly shorter than *d*_H–M_ (2.02 *vs*. 2.30 Å), indicating a strong deformation upon benzene adsorption. Based on the PES and adsorption heights, we conclude the chemisorptive character for benzene on the Cu(110) surface.

Different from Cu(110), the nature of bonding for benzene on Ag(110) and Au(110) surfaces are predominated by physisorptive character, as evidenced by the weaker binding energies and larger adsorption heights (Fig. 1b and c). For benzene on Ag(110), the largest difference in binding energies is 0.17 and 0.14 eV from PBE+MBD and PBE+vdW^surf^, respectively. The c2 structure is the most stable structure from both PBE+MBD (−0.67 eV) and PBE+vdW^surf^ (−0.82 eV). On the other hand, c6 is the most preferable structure for benzene/Au(110) from both PBE+MBD (−0.75 eV) and PBE+vdW^surf^ (−0.86 eV). The c5 structure, which is the most stable structure reported by Matos *et al*.^37^, is only 10 meV less than c6 from PBE+MBD. Figure 1b also shows that the energy corrugations for benzene on Cu(110) and on Au(110) are close to each other (0.24–0.25 eV), being evidently larger than that for Ag(110) (0.17 eV from PBE+MBD). This result further suggests that Ag(110) is more inert than its Cu and Au counterparts. In addition, the relatively larger and almost identical adsorption distances for C and H above metals clearly indicate the physisorptive character for benzene on the Ag(110) and Au(110) surfaces.

**Figure 2:**
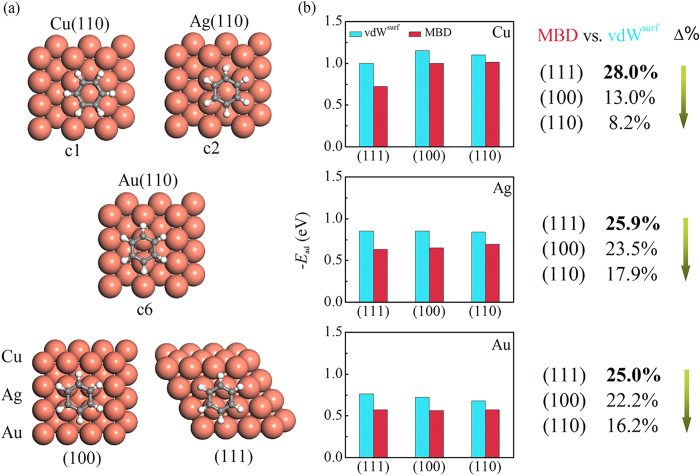
(**a**) The preferred adsorption structures for benzene on coinage metal surfaces. (**b**) The dispersion contributions for benzene adsorption systems. Note that the energetics were obtained by decomposing the PBE+MBD and PBE+vdW^surf^ adsorption energies into the local and non-local correlation (vdW^surf^ or MBD) contributions. Δ% = 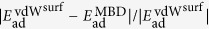, which are closely related to the planar atomic density of FCC crystals.

Having explored the PES for (110) surfaces, we now continue to study the interactions of benzene with the (100) and (111) surfaces of coinage metals. We would not systematically search the PES for the (100) and (111) surfaces because (1) they have been more extensively studied, in particular for the (111) surfaces; and (2) no uncertainties exist about their most stable adsorption sites. For benzene/(100), Chen *et al*.^38^ have considered six possible adsorption sites (termed as “hollow”, “hollow-15”, “bridge”, “bridge-30”, “top”, and “top-15”) at the Au(100) surface, and reported that the hcp hollow site is preferable. The same finding has also been reached by Reckien *et al*.^21^ from the DFT-D3 method. As such, we directly relaxed the adsorption structures at the hollow site for benzene on the (100) surfaces of Cu, Ag, and Au (Fig. 2a).

For benzene on Cu(111) surfaces, our recent work has systematically studied eight starting geometries for benzene on coinage metal (111) surfaces, and achieved a flat PES for all physisorbed systems^22^. These observations are in excellent agreement with STM experiments^39^,^40^,^41^, which demonstrated that benzene molecules can diffuse almost freely over these surfaces at low temperatures. For consistency in the present work we still use the bridge-30 site for benzene on the (111) surfaces (Fig. 2a).

Table 1 shows the benzene adsorption energies and heights at the most stable structures computed by PBE+vdW^surf^ and PBE+MBD. The MBD effects are found to contribute differently on surfaces with distinct planar atomic density. More specifically, the adsorption energy for benzene on Cu(111) is the smallest among all three Cu surfaces from PBE+vdW^surf^ (−0.89 eV) and PBE+MBD methods (−0.63 eV). Adsorption energies decrease in the order of Cu(110) > Cu(100) > Cu(111), and inversely, adsorption heights increase as Cu(110) < Cu(100) < Cu(111). This trend can be well explained by the increasing coordination number of the surface metal atoms: the larger the metal coordination number of the surface atom, the more reactive the surface^35^. For benzene on Cu(111), the adsorption energy from PBE+MBD method decreases by 0.26 eV compared with the PBE+vdW^surf^ value. While on the Cu(100) and (110) surfaces, the adsorption energy decreases by 0.15 and 0.10 eV, respectively.

To understand the origin of adsorption energy difference, we decompose the total adsorption energy of the system into the local and the non-local contributions. As shown in Fig. 2b, compared to the vdW^surf^ part from the PBE+vdW^surf^ method, the adsorption energy of MBD part from the PBE+MBD method is reduced by 0.28 eV (28%) for benzene on Cu(111). While on Cu(100) and (110), the adsorption energy of MBD part decreases by 0.15 eV (13%) and 0.09 eV (8.2%), respectively. Therefore, MBD effects contribute mostly to the closest-packed Cu(111) surface, but leastly to the most sparse Cu(110) surface. Importantly, we find that the sequence of MBD effects is in line with the planar atomic density of FCC surfaces, which are 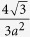 for (111), 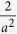 for (100), and 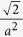 for (110), where *a* is the lattice constant of FCC crystal. The same trend can also be achieved for benzene adsorbed on the low-index surfaces of Ag and Au, see Fig. 2.

Table 1 shows an interesting finding that the PBE+MBD binding energy for benzene/Au(110) is 80 meV larger than that for benzene/Ag(110). This result seems to contradict the intuition that Ag surfaces are more reactive (or at most equal stable) than Au surfaces. To understand this, we computed the polarizability of adsorption systems and found that the polarizability of Au (5.61 bohr^3^ along the perpendicular direction to the slab) is significantly larger than that of Ag (4.47 bohr^3^) upon benzene adsorption. This agrees with the assumption by Reckien *et al*.^21^, which addressed that the higher reactivity of Au compared to Ag is attributed to the larger polarizability of the Au atoms. However, inconsistant with ref. 21, our results show that the dispersion energy for benzene on Ag(110) is 0.12 eV larger than that on Au(110). Interestingly, although single metal atom cannot represent the surface, the accurate CCSD(T) functional also found that the binding energy for Au/benzene cluster is stronger than that for Ag/benzene cluster^42^. Using the same cluster models reported in the CCSD(T) calculations, our PBE+vdW calculations also demonstrate this finding (−0.14 *vs.* −0.31 eV for benzene on a single Ag and single Au atom, respectively). We find that Au/benzene cluster is indeed more reactive than Ag/benzene cluster, consistent with our results for benzene/Au(111) and benzene/Ag(111).

**Figure 3:**
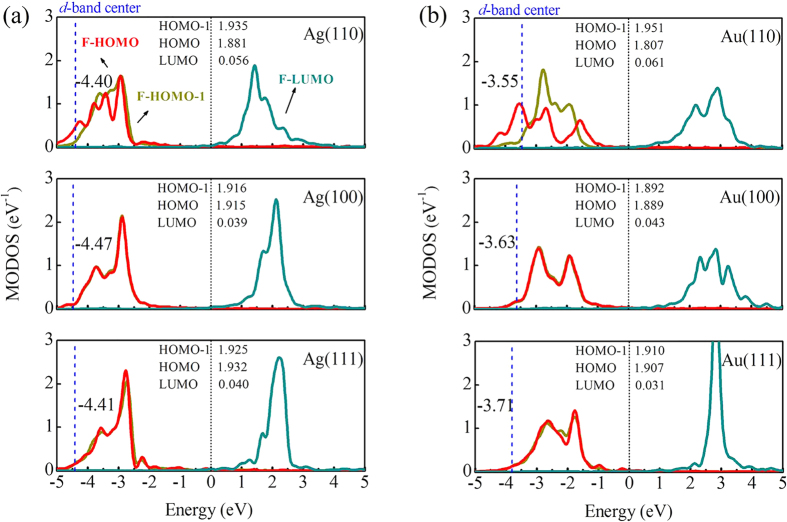
MODOS projected on the free benzene molecule HOMO-1, HOMO, and LUMO orbitals (F-HOMO-1, F-HOMO, and LUMO) for benzene on Ag and Au surfaces. The zero of energy corresponds to the Fermi level. The blue dashed line represents the *d*-band center of metal surfaces. The center of gravity of HOMO-1 and HOMO orbitals for benzene/Ag(110) is at −3.29 and −3.44 eV, respectively. On the other hand, the center of HOMO-1 and HOMO is located at −2.56 and −2.89 eV on Au(110), respectively.

The above “abnormal” energy sequence for benzene on Ag(110) and Au(110) surfaces can be further explained by the computed projected densities of states. As shown in Fig. 3, the adsorption of benzene on Ag(110) and Au(110) breaks the degeneration of its HOMO and HOMO-1 orbitals of the adsorbate benzene, due to the strong perturbation of the metal substrates. We also calculated the center of gravity of HOMO-1 and HOMO orbitals for benzene/Ag(110) and benzene/Au(110) systems. In the former case, the center of HOMO-1 and HOMO of the adsorbate is at −3.29 and −3.44 eV, respectively. On the other hand, the center of HOMO-1 and HOMO is located at −2.56 and −2.89 eV on Au(110), respectively. Importantly, the difference of center between HOMO-1 and HOMO of Au(110) (0.33 eV) is twice larger than that of Ag(110) (0.15 eV). We thus conclude that the larger splitting of HOMO and HOMO-1 orbitals of Au(110) leads to the larger adsorption energy than Ag(110). Analysis of the projected orbitals occupation indicates that for Ag(110) there are 1.881 electrons in the HOMO orbital, which is larger than the corresponding value of the Au(110) surface (1.807 electrons). Meanwhile, the occupation number of the LUMO state is slightly smaller for benzene on Ag(110) than for that on Au(110) [0.056 *vs.* 0.061 electrons]. In contrast to (110), the HOMO and HOMO-1 orbitals for (100) and (111) surfaces are still degenerate, which indicates a small amount of charge transfer. Overall, the splitting/broadening of the HOMO and HOMO-1 orbitals provides the stronger binding energy for benzene on Au(110) than Ag(110). Note that, the PBE functional would underestimate the band gap due to the self-interaction error, especially for free molecules^43^. However, the conclusions obtained based on molecular orbital analysis would not be qualitatively changed for most adsorption systems^44^,^45^,^46^.

The above conclusion, *i.e.* Au(110) surface is more reactive than Ag(110), is found to be a general message for aromatic molecules. As shown in Table 2, the computed adsorption energy for benzene, naphthalene, and anthracene on Au(110) is 80, 150, and 300 meV larger than that on Ag(110) from PBE+MBD method, respectively. Note that, the adsorption energy difference between Au and Ag is mainly originated from the PBE part, in the range of 0.20–0.38 eV for benzene, naphthalene, and anthracene. In addition, the dispersion energies for molecules on Ag(110) are 0.08–0.17 eV larger than those on Au(110), which is attributed to the smaller *screened* vdW radius for Ag atoms (2.57 *vs.* 2.91 bohr for Ag and Au)^22^.

Finally, we determined the *d*-band center for each system to understand their energetics. The *d*-band center theory was first proposed by Hammer and Nørskov, with the aim to understand the trends in reactivity of H_2_ outside transition and noble metals and their alloys^47^. It has been shown that the *d*-band model can predict the trends in chemisorption energies of various small adsorbates on metal surfaces^47^,^48^,^49^. For different metal surfaces with the same Miller index, the theory addresses that the higher the *d*-band center, the more reactive the metal. Indeed, for the (110) surface the *d*-band center of Au is significantly closer to the Fermi level than that of Ag, implying the stronger interactions of benzene with Au(110) (see Fig. 3). However, this theory apparently against what we observed for benzene on (111) and (100) surfaces. In this case, although the *d*-band center of Au remains much closer to the Fermi level than Ag, the binding energies for benzene on Ag(111) and Au(111) are almost identical, due to the delicate balance between the Pauli repulsion and relatively larger vdW energies in these systems. Finally, we noticed that the pure PBE data for the (111) and (100) surfaces still obey the *d*-band center theory (Table 1). Therefore, it is most likely that the larger amount of vdW contributions can change the energy hierarchy, and in turn, leads to the “failure” of the theory for benzene on the (111) and (100) surfaces.

## Discussion

In summary, we have used the many-body dispersion method to systematically study benzene on the low-index coinage metal surfaces. We demonstrate that the many-body contributions are sensitive to the planar atomic density of FCC metals, and follow the sequence of (111) > (100) > (110). Our calculations show that the binding energy for benzene on Au(110) is even larger than that on Ag(110), due to the larger broadening and splitting of benzene molecular orbitals (HOMO and HOMO-1) that lie just below the Fermi level. The delicate balance between Pauli repulsion and vdW forces leads to the identical binding energy for benzene on Ag(111) and on Au(111), which goes against the classic *d*-band center theory that works well for small molecules on metals.

## Methods

We performed DFT calculations using the semi-local PBE^36^ functional with either the vdW^surf ^34 or many-body dispersion (MBD)^25^,^26^ methods for including vdW interactions. The PBE+vdW^surf^ is based on a pairwise atom-atom approximation and includes electrodynamic screening of vdW interactions by combining intermolecular vdW interactions with the Lifshitz-Zaremba-Kohn theory^50^ for the dielectric screening within the metal surface. In contrast to the vdW^surf^ method, the MBD method is based on the many-body dispersion effects. PBE+vdW^surf^ and PBE+MBD calculations were carried out using the numeric atom-centered basis set all-electron code FHI-aims^51^,^52^ applying a scaled zeroth-order regular approximation (ZORA) for treating relativistic effects. For all computations, we used “tight” settings and set the following thresholds for the convergence criteria: 10^−2^ eV/Å for the final forces in all structural relaxations, 10^−5^ electrons for the electron density, and 10^−4^ eV for the total energy of the system. The metal lattice constants used here are Cu (3.572 Å), Ag (4.007 Å) and Au (4.163 Å), which are calculated with the PBE+vdW^surf^ method^22^. For the adsorption of benzene, the (110), (100), and (111) metal surfaces was modeled by periodic (3 × 4), (4 × 4), and (4 × 4) unit cells respectively, containing six atomic layers separated by at least 100 Å of vacuum. For the adsorption of naphthalene and anthracene, Ag(110) and Au(110) surfaces were modeled by periodic (3 × 4) and (4 × 4) unit cells, respectively. Note that all metal surfaces studied in the present work are unreconstructed. The aromatic molecules and the uppermost two metal layers were allowed to relax during geometry relaxation. The four bottom metal layers were fixed at their bulk-truncated positions using the lattice constants of the bulk metals from PBE+vdW^surf^ method. We carried out systematic convergence tests and calculated the binding energy and adsorption height for the benzene/Cu(100) system using (5 × 5) supercell and 10 metal layers, respectively. Further increasing the unit cell from (4 × 4) to (5 × 5) only leads to 30 meV difference in adsorption energy. Similarly, increasing the slab number from 6 to 10 only leads to 30 meV difference in adsorption energy and 0.04 Å. difference in adsorption height.

## Additional Information

**How to cite this article**: Jiang, Y. *et al*. Aromatic molecules on low-index coinage metal surfaces: Many-body dispersion effects. *Sci. Rep.* **6**, 39529; doi: 10.1038/srep39529 (2016).

**Publisher's note:** Springer Nature remains neutral with regard to jurisdictional claims in published maps and institutional affiliations.

## Figures and Tables

**Table 1:** Computed binding energies and adsorption heights for benzene on low-index surfaces of coinage metals at the most preferable sites.

Systems	*E*_ad_ (eV)					*d* (Å)		Δ (eV)
	PBE+vdW^surf^	PBE+MBD	PBE	MBD	Exp.	*d*_C–M_	*d*_H–M_	
Cu(111)	−0.89	−0.63	0.10	−0.73	−0.69 ± 0.04	2.83	2.82	0.26
Cu(100)	−1.20	−1.05	−0.05	−1.00	—	2.22	2.41	0.15
Cu(110)	−1.20	−1.10	−0.10	−1.00	—	2.02	2.30	0.10
Ag(111)	−0.79	−0.57	0.06	−0.63	−0.68 ± 0.05	2.97	2.95	0.22
Ag(100)	−0.81	−0.62	0.04	−0.66	—	2.91	2.91	0.19
Ag(110)	−0.82	−0.67	0.02	−0.69	—	2.75	2.74	0.15
Au(111)	−0.75	−0.56	0.01	−0.57	−0.65 ± 0.03	3.05	3.04	0.19
Au(100)	−0.81	−0.65	−0.09	−0.56	—	2.96	2.97	0.16
Au(110)	−0.86	−0.75	−0.18	−0.57	—	2.71	2.73	0.11

For comparison the available experimental data^33^ are also shown in the table. The PBE and MBD values are obtained based on the PBE+MBD adsorption energies. Δ is the adsorption energy difference between PBE+vdW^surf^ and PBE+MBD methods, which is defined as Δ = |*E*_ad_(PBE+vdW^surf^) − *E*_ad_(PBE+MBD)|.

**Table 2:** Computed binding energies and adsorption heights for benzene, naphthalene, and anthracene molecules on Ag(110) and Au(110) surfaces at the most preferable sites.

Systems	*E*_ad_ (eV)					*d* (Å)	
	PBE+vdW^surf^	PBE+MBD	PBE	vdW^surf^	MBD	*d*_C–M_	*d*_H–M_
Benzene/Ag(110)	−0.82	−0.67	0.02	−0.84	−0.69	2.75	2.74
Benzene/Au(110)	−0.86	−0.75	−0.18	−0.68	−0.57	2.71	2.73
Naphthalene/Ag(110)	−1.28	−1.04	−0.03	−1.25	−1.01	2.74	2.76
Naphthalene/Au(110)	−1.36	−1.19	−0.26	−1.10	−0.93	2.69	2.75
Anthracene/Ag(110)	−1.93	−1.66	−0.17	−1.76	−1.49	2.59	2.67
Anthracene/Au(110)	−2.14	−1.96	−0.55	−1.59	−1.41	2.50	2.64

The PBE, vdW^surf^, and MBD values are obtained based on the PBE+vdW^surf^ and PBE+MBD adsorption energies.
